# Association of mitochondrial haplogroup F with physical performance in korean population

**DOI:** 10.5808/GI.2019.17.1.e11

**Published:** 2019-03-31

**Authors:** In Wook Hwang, Kicheol Kim, Eun Ji Choi, Han Jun Jin

**Affiliations:** 1 Department of Biological Sciences, College of Natural Science, Dankook University, Cheonan 31116, Korea; 2 Department of Neurology, Weill Institute for Neurosciences, University of California San Francisco, San Francisco, CA 94158, USA

**Keywords:** athletes, haplogroup, Korean population, mitochondrial DNA, physical functional performance

## Abstract

Athletic performance is a complex multifactorial trait involving genetic and environmental factors. The heritability of an athlete status was reported to be about 70% in a twin study, and at least 155 genetic markers are known to be related with athlete status. Mitochondrial DNA (mtDNA) encodes essential proteins for oxidative phosphorylation, which is related to aerobic capacity. Thus, mtDNA is a candidate marker for determining physical performance. Recent studies have suggested that polymorphisms of mtDNA are associated with athlete status and/or physical performance in various populations. Therefore, we analyzed mtDNA haplogroups to assess their association with the physical performance of Korean population. The 20 mtDNA haplogroups were determined using the SNaPshot assay. Our result showed a significant association of the haplogroup F with athlete status (odds ratio, 3.04; 95% confidence interval, 1.094 to 8.464; p = 0.012). Athletes with haplogroup F (60.64 ± 3.04) also demonstrated a higher Sargent jump than athletes with other haplogroups (54.28 ± 1.23) (p = 0.041). Thus, our data imply that haplogroup F may play a crucial role in the physical performance of Korean athletes. Functional studies with larger sample sizes are necessary to further substantiate these findings.

## Introduction

Athletic performance is a complex multifactorial trait affected by genetic and environmental factors [1]. Genetic factors are particularly known to contribute to strength, endurance, power, and aerobic capacity [2]. A twin study reported the heritability of an athlete status as 70% [3]. To date, at least 155 genetic markers are known to be related to the athlete status [2]. *ACE* gene is the first reported genetic marker associated with athletic performance and known to regulate vasoconstriction [4]. Previous studies reported an association between ACE enzyme activity and endurance performance [5, 6]. *ACTN3* is the most studied genetic marker; the XX genotype of *ACTN3* R577X polymorphism has a complete deficiency of α-actinin-3 [7]. Several studies reported the association of the RR genotype of *ACTN3* with sprint performance, whereas the XX genotype reportedly contributed to endurance performance [7-10]. However, despite several genetic approaches, the effect of genetic markers on athletic performance has not yet been fully understood [2].

Aerobic capacity reportedly plays an important role in endurance performance [11]. Additionally, aerobic capacity has been shown to be inherited maternally rather than paternally [12]. Mitochondria are subcellular organelles that generate 36 molecules of adenosine triphosphate (ATP) by oxidative phosphorylation (OXPHOS), whereas two ATP molecules are produced through glycolysis [13]. Mitochondria have their own maternally inherited circular DNA (mtDNA), which is approximately 16,569 bp in size. It encodes 13 OXPHOS, two *rRNA*, and 22 *tRNA* genes [14]. Because mtDNA is haploid, it does not undergo recombination [15]. Therefore, the human population can be defined as a pool of various haplogroups based on accumulated specific mtDNA polymorphisms, with the polymorphism frequencies differing between populations [16, 17]. Several studies have been conducted in various populations to identify the role of population-specific mtDNA haplogroups on the expression of phenotypes including diseases and longevity [11, 18, 19].

The sprint and endurance ability of athletes is determined by different genetic factors [20]. Aerobic capacity is necessary for endurance performance and is regulated by mitochondrial OXPHOS. Thus, it is believed that mitochondria play an important role in endurance performance [21, 22]. Initial studies used familial studies to report on associations between mtDNA and exercise phenotype [23, 24]. A number of genetic studies have been performed using the mtDNA haplogroup for athlete status and physical performances. The haplogroup H has been reportedly related to higher oxygen consumption and has been associated with athlete status in Finnish and Polish populations [20, 25]. Scott et al. (2009) [26] reported that haplogroup L0 (African specific haplogroup) contributes to physical performance in Kenyan population. Furthermore, the association of haplogroups G1 and F with athlete status in Japanese population has also been reported [27]. Haplogroups M and N9 are involved with athlete status in Korean population [17]. Together, these results suggest that mtDNA haplogroups may play an important factor in athlete status and/or physical performance.

The frequency of mtDNA haplogroups vary among ethnic groups, mainly to differing different genetic backgrounds and environmental factors [12]. This suggests that multiple replication studies are necessary to clarify the role of mtDNA haplogroup in athlete status among independent ethnic groups. Therefore, from Korean population, we recruited a total of 256 college-level subjects (111 athletes and 145 controls) and analyzed the genetic association between 20 mtDNA haplogroups and athlete status as well as physical performance.

## Methods

### Subjects

We analyzed a total of 111 athletes (85 males and 26 females) enrolled in College of Sports Science at Dankook University in Cheonan, Korea (Table 1). The athlete group included subjects who participated in basketball, climbing, rugby, soccer, golf, baseball, ssireum, rowing, speed skating, short track, soft ball, tennis, soft tennis, marathon, running, judo, badminton, swimming, horse-riding, weight lifting, aerobics, jazz dance, body building, rhythmic gymnastics, squash, taekwondo, shooting, and futsal. The control group involved a total of randomly selected (therefore, likely to be unrelated) 145 subjects (72 males, 73 females) among students of College of Natural Science at Dankook University; none were regularly trained for athletics or had success in any official competitions. The study was approved by the Ethics Committee and Institutional Review Board of Dankook University, Korea and conformed to the standards set by the Declaration of Helsinki. A separate written informed consent was obtained for enrolment in the study from all the subjects.

### Physical fitness tests

Seven performance tests were carried out. These included 20 m shuttle run, Sargent jump, right and left hand grip, 50 m run, sit-up, side-step, and sit-and-reach. We measured the fitness tests only for possible participants due to the long measuring time, so some of the fitness tests results were missing. The test protocols are described below.

- 20 m shuttle run: The subjects were required to run back and forth between two lines set 20 m apart. Running pace was regulated by a sound signal. The starting speed was set to 8.5 km/h and increased by 0.5 km/h every minute. The test ended when the subjects failed to reach the target line in time.

- Sargent jump: Test subjects were made to stand on a platform and were belted at the waist. Next, they were made to jump vertically as high as possible using both arms and legs. The measurement rope was then pulled onto the platform when the subjects jumped vertically and the length of the rope was compared to each other.

- Hand grip: Hand grip was assessed using Takei A5401-Digital Hand Grip Strength Dynamometer (Takei, Yashiroda, Japan). The subjects spread out their arms while squeezing as forcefully as possible (right, left), palms facing inward.

- 50 m run: The subjects were made to stand at a starting line and then made to run forward at full speed for a distance of 50 m.

- Sit-up: Subjects were made to sit on mats with knees bent at an angle of 90° with hands placed on both sides of the head. One sit-up was determined as touching the knees with the elbows and returning the hands to the ground. The number of sit-ups performed within one minute was counted.

- Side-step: Subjects were made to stand on a ground with a mid-line, which was equidistantly marked (100 cm) on either side with a parallel line each. They were made to step to the right until their right foot reached the right-line. Next, they were made to step to the left-line and pass the mid-line. After left foot reached the left-line subjects returned to their original position on the mid-line. This process was repeated for 60 s.

- Sit-and-Reach: Subjects were made to sit on a mat. Their knees pointed upwards as they stretched out legs. Next, they were made to bend forward with their upper body with their hands outstretched to push the measuring instrument.

### DNA extraction and genotyping

DNA was extracted from buccal swabs using the GeneAll Exgene Clinic SV mini kit (GeneALL, Seoul, Korea). We analyzed a total of 20 mtDNA haplogroups specific to East Asia from previous report (M, D, D4, D4a, D4b, D4b2, D5, M7, M8, M9, M10, M11, G, C4, N9a, Y, A, F, B4, and B5) [28]. For mtDNA haplogroups determination, we used a 20-plex SNaPshot assay [28]. Primer sequences for polymerase chain reaction (PCR) amplification and single base extension (SBE) reaction were presented in Supplementary Tables 1 and 2 [28]. The PCR reaction was performed in a total volume of 10 μL containing 10 ng DNA of genomic DNA, 0.035–0.180 μM each primer, 0.2 mM dNTPs, 1× PCR buffer, 4 mM MgCl_2_, and 0.5 U AmpliTaq Gold DNA polymerase (Applied Biosystems, Foster City, CA, USA) on a C1000 Touch thermal cycler (Bio-Rad, Hercules, CA, USA). The cycling conditions were 95℃ for 10 min followed by 35 cycles of amplification at 94℃ for 15 s, 60℃ for 15 s, 72℃ for 30 s, and a final extension at 70℃ for 10 min. PCR products were purified by adding 1 μL ExoSapIT (USB, Santa Clara, CA, USA). Twenty-plex SBE reaction was performed on a C1000 Touch thermal cycler (Bio-Rad) in 6 μL with 1 μL of purified PCR product, 1.75 μL of SBE primer mix (0.04–0.18 μM of each primer), and 1 μL of SNaPshot reaction mix (Applied Biosystems). The cycling conditions: 25 cycles of 96℃ for 10 s, 50℃ for 5 s, and 60℃ for 30 s. The remaining fluorescent ddNTPs were removed by addition of 1 U shrimp alkaline phosphatase to the SBE reaction product and incubation at 37℃ for 45 min followed by 80℃ for 15 min. Samples were analyzed by capillary electrophoresis on an ABI Prism 310 Genetic Analyzer in which 1 μL of SBE product was mixed with 14 μL of Hi-Di formamide and 0.4 μL of GeneScan 120 LIZ internal size standard. Automated allele calls were made using GeneMapper v.4.0.

### Data analyses

Mann–Whitney U-test was performed using the SPSS version 21 Statistics software (IBM Corp., Armonk, NY, USA) to test the significance of differences in continuous variables between the mitochondrial haplogroup F and results from the physical performance tests. Multivariate logistic regression analysis was used to adjust the distribution of gender between athletes and controls. The test of cross tabulation analyses and odds ratio (OR) with 95% confidence intervals (CI) in a 2 × 2 table was calculated using a statistical analysis program available on the internet (SISA, http://www.quantitativeskills.com/sisa). Since mtDNA is haploid, the markers of mtDNA are in linkage disequilibrium. Since haplogroup comparisons are not independent, the adjustment of *p*-values for mtDNA haplogroups is not recommended for multiple testing [29]. In line with this literature reported recommendation, the *p*-values for multiple testing were not adjusted.

## Results

Our result showed that the mtDNA haplogroup F was significantly more frequent in athletes (12.6%) than in the control group (4.1%) (OR, 3.34; 95% CI, 1.241 to 9.007; p = 0.012) (Table 2). In addition, multivariate logistic regression analysis was performed to adjust the confounding factor (sex); the adjusted OR and 95% CI for the mtDNA haplogroup F was 3.04 and 1.094–8.464, respectively (Table 2). We also performed the correlation analysis to identify a relationship between the physical performances and haplogroup F. Here, we found that athletes with haplogroup F (60.64 ± 3.04) showed a higher Sargent jump than athletes with other haplogroups (54.28 ± 1.23) (p = 0.041).

## Discussion

The frequencies of mtDNA haplogroups analyzed in our study are summarized in Table 2. We observed significant difference in mtDNA haplogroup F between athletes and controls (p < 0.05). The frequency of mtDNA haplogroup F in the control group was consistent with those of the previous study [30]. The haplogroup-determining polymorphisms for haplogroup F are 249delA, C3970T, T6392C, G10310A, and G13928C, in which 249delA is located in the noncoding region (D-loop) and the other three variants are positioned in the coding region [27, 31]. Among the polymorphisms of the coding region, only G13928C is a non-synonymous variant that causes a Ser531Thr replacement in *ND5*. *ND5* encodes the NADH-ubiquinone oxidoreductase chain 5 protein, a subunit of NADH dehydrogenase that is a part of complex I for OXPHOS [27]. Previous studies have shown that the haplogroup F exhibits a low complex I activity in mitochondria and is associated with type 2 diabetes [18, 32]. Other studies reported haplogroup F to be a protective factor against various traits including Leber’s hereditary optic neuropathy, hearing loss, and aging [16, 33, 34]. Thus, haplogroup F can play a functional role in the expression of various phenotypes including athlete status.

A genetic association between physical performance and mtDNA haplogroup F was also analyzed (Table 3). Here, we found that athletes with haplogroup F (60.64 ± 3.04) showed a higher Sargent jump than athletes with other haplogroups (54.28 ± 1.23) (p = 0.041). Sargent jump is a typically known test for measuring power [35]. This result is different from those observed in previous studies suggesting that mitochondria are mainly related to endurance performance [21, 22]. Interestingly, several studies reported that a few specific mtDNA haplogroups are associated with sprint performance [22, 27]. Fuku et al. (2012) [22] found that the macrohaplogroup N contributes to stronger leg extension power and higher vertical jump in Japanese adults (p < 0.05). Mitochondria regulate the intracellular calcium dynamics, which contribute to muscle contraction [36]. In this context, it has been previously reported that the macrohaplogroup N exhibits higher calcium levels in mitochondria compared with the macrohaplogroup M [37]. This indicates that the macrohaplogroup N may influence anaerobic performance such as muscle power. The mtDNA haplogroup F is a subhaplogroup of the macrohaplogroup N (http://www.phylotree.org). Meanwhile, Mikami et al. (2011) [27] recruited 139 Olympic athletes (79 endurance/middle-power athletes, 60 sprint/power athletes) to understand the genetic correlations between mitochondrial haplogroup and elite Japanese athletes. This study observed that the frequency of mtDNA haplogroup F in sprint/power athletes was higher than in the control group (OR, 2.79; 95% CI, 1.28 to 6.07; p = 0.007) [27]. The authors emphasized the role of haplogroup F in inducing glycogen breakdown, which is then used as a fuel for glycolysis required for skeletal muscle contraction. Furthermore, anaerobic capacity (sprint performance) prefers the glycolytic pathway compared with OXPHOS to acquire ATP [22]. Thus, our result supports a previous finding. However, further replicative studies in various populations are warranted.

A limitation of the current study was the relatively small sample size. The sample power of association analysis in our study was 73%. The ideal sample power of association analysis is reported to be approximately 80% [38]. Therefore, larger sample sets are required to further clarify the role of mtDNA haplogroup F on athlete status. Also, the athlete group consisted of more than 27 sports categories, and the different physical abilities of each athlete would be cause for the heterogeneity of physical performance. Additionally, we recruited college-level athletes who were not national elite athletes. However, they were regularly trained to win official competitions.

Our study presents the following advantage: a positive association between the haplogroup F and athlete status was reported only in case of Japanese population. To the best of our knowledge, our results are the first to replicate a previous finding and hence can establish an important subject for meta-analyses as well as for further studies.

In conclusion, our results imply that the haplogroup F may have a significant effect on athlete status and sprint performance in Korean population. However, larger sample sizes and functional studies are necessary to further elucidate our findings.

## Figures and Tables

**Table 1.** Subject characteristics of Korean sport players and control groups

Characteristic	Athlete (n=111)	Control (n=145)	p-value
Sex			
Male	85	72	<0.001
Female	26	73	
Age, mean ± SD (yr)	21.1 ± 2.26	21.3 ± 2.54	0.858
Sport			
Basketball	9	-	
Climbing	2	-	
Rugby	14	-	
Soccer	18	-	
Golf	1	-	
Baseball	15	-	
Ssireum	3	-	
Rowing	4	-	
Speed skating	1	-	
Short track	1	-	
Softball	4	-	
Tennis	2	-	
Soft tennis	1	-	
Marathon	1	-	
Running	3	-	
Judo	3	-	
Badminton	9	-	
Swimming	2	-	
Horse-riding	2	-	
Weightlifting	5	-	
Aerobics	2	-	
Jazz dance	1	-	
Bodybuilding	2	-	
Rhythmic gymnastics	1	-	
Squash	2	-	
Taekwondo	1	-	
Shooting	1	-	
Futsal	1	-	

**Table 2.** Association between athlete status and individual mtDNA haplogroup in this study

Haplogroup	Athlete (n = 111)				Control (n = 145)
						No. of samples (%)	OR (95% CI)		p-value^a^
F	14 (12.6)	3.04 (1.094–8.464)^b^		0.012^*^	6 (4.1)
N9a	7 (6.3)	1.89 (0.582–6.105)		0.219	5 (3.4)
G	6 (5.4)	1.13 (0.368–3.451)		0.526	7 (4.8)
D4a	5 (4.5)	1.74 (0.587–5.163)		0.229	11 (7.6)
B4	15 (13.5)	1.26 (0.594–2.673)		0.339	16 (11.0)
D4	20 (18.1)	1.34 (0.721–2.493)		0.221	33 (22.8)
A	9 (8.1)	1.51 (0.644–3.517)		0.231	17 (11.7)
D5	7 (6.3)	1.02 (0.367–2.821)		0.586	9 (6.2)
N9	0	-		1.000	0
M7	11 (9.9)	1.12 (0.480–2.597)		0.481	13 (9.0)
B5	4 (3.6)	1.36 (0.387–4.756)		0.439	7 (4.8)
M10	3 (2.7)	0.25 (0.026–2.437)		0.218	1 (0.7)
M9	2 (1.8)	0.38 (0.034–4.228)		0.401	1 (0.7)
M8	5 (4.5)	1.24 (0.394–3.893)		0.474	8 (5.5)
Y	1 (0.9)	0.43 (0.044–4.194)		0.417	3 (2.1)
M	1 (0.9)	-		0.434	0
D	0	-		1.000	0
N	0	-		1.000	0
D4b	1 (0.9)	0.18 (0.022–1.479)		0.070	7 (2.8)
M11	0	-		0.500	1 (0.7)
					

mtDNA, mitochondrial DNA; OR, odds ratio; CI, confidence interval.

* Significant association: p < 0.05.

a Uncorrected chi-square or Fisher exact test, as appropriate;

b OR adjusted in multivariate logistic regression model including gender and mtDNA haplogroup F.

**Table 3.** Association between the physical performances and mtDNA F haplogroup

Fitness test	mtDNA haplogroup				p-value^a^
	F		Other		
	No.	Mean ± SD	No.	Mean ± SD	
20 m shuttle run	10	74.50 ± 29.62	63	64.25 ± 34.69	0.380
Single leg stance (R)	13	28.94 ± 21.51	96	34.85 ± 25.25	0.422
Single leg stance (L)	7	35.52 ± 19.16	62	39.87 ± 22.30	0.622
Sagent jump	14	60.64 ± 3.04	96	54.28 ± 1.23	0.041^*^
Hand grip (R)	14	44.34 ± 10.61	103	41.18 ± 10.67	0.300
Hand grip (L)	14	40.89 ± 11.00	102	39.14 ± 11.37	0.589
50 m running	12	6.89 ± 0.69	77	8.33 ± 5.48	0.366
Sit-up	14	44.93 ± 12.38	99	45.60 ± 11.98	0.846
Side-step	13	59.69 ± 7.35	85	62.40 ± 24.41	0.693
Sit-and-Reach	14	26.18 ± 7.78	101	24.46 ± 15.30	0.680

mtDNA, mitochondrial DNA.

* Significant association: p < 0.05.

a Mann–Whitney U test.

## Supplementary Materials

Supplementary Table 1. Primers for polymerase chain reaction amplification

Supplementary Table 2. Primers for single base extension reaction
