# Supplementary material for: Association of mitochondrial haplogroup F with physical performance in korean population
Source: Genomics Inform. 2019 Mar 31;17(1):e11. doi: 10.5808/GI.2019.17.1.e11 (PMC6459174; doi:10.5808/GI.2019.17.1.e11)
Supplement: Supplementary Table 1. — Primers for polymerase chain reaction amplification [file gi-2019-17-1-e11-suppl1.pdf]

Supplementary Table 1. Primers for polymerase chain reaction amplification

Haplogroup	Motif	Start	Forward	Tm (°C)	Start	Reverse	Tm(°C)	Size(bp)
M, D5	A10397G	10358	AAGTCTGGCCTATGAGTGAC	57.1	10481	TGTAAATGAGGGGCATTTGG	56.4	128
F	G10310A	10273	TTTTACCCCTACCATGAGCC	56.5	10356	GGGCTAGGATGATGATTAATAAGA	56.3	84
D, N9a	C5178a	5146	CGACCCTACTACTATCTCGC	56.9	5280	TGAATTCTTCGATAATGGCCC	56.6	135
	G5231A							
D4	G3010A	2929	CTAGGGATAACAGCGCAATC	56.6	3038	AACGAACCTTTAATAGCGGC	57	110
M7	T9824C	9742	CAGAGTACTTCGAGTCTCCC	57	9867	GGATGAAGCAGATAGTGAGGA	57.1	126
M8	A15487t	15434	CTCGGCTTACTTCTCTTCCT	56.9	15529	GGGGTTGGCTAGGGTATAAT	56.5	96
M9	G4491A	4430	CCCATACCCCGAAAATGTTG	58.3	4571	GCCTGCAAAGATGGTAGAGT	59.3	88
M10, A	C8794T	8761	ATTGCCACAACTAACCTCCT	57.2	8841	GGCCATGGCTAGGTTTATAG	55.8	81
M11	G11969A	11893	ACTCTCTGTGCTAGTAACCAC	58.3	12013	TGAGCCCCATTGTGTTGTG	60.1	121
G	A4833G	4791	ATAGCCCCCTTTCACTTCTG	57.4	4927	AGGCTTACGTTTAGTGAGGG	57.4	137
Y	T14178C	14156	CCCCGAGCAATCTCAATTAC	56.8	14296	TTATGAAGGAGAGGGGTCAG	56.1	141
B4,5	8281-8289d	8194	CCACAGTTTCATGCCCATC	57	8310	AGTTAGCTTTACAGTGGGCT	56.9	117
D4a	T14979C	14946	CCCACATCACTCGAGACG	58.7	15064	TGATCCGTAATATAGGCCTCG	59.1	119
N	C10873T	10827	TTTGAATCAACACAACCACCC	58.7	10925	AGGTTGGGGAACAGCTAAAT	58.7	99
N9	G5417A	5352	CGCCTAATCTACTCCACCTC	58.4	5442	ATGGGGTGGGTTTTGTATGT	59.1	91
D4b	G8020A	7922	TACGGCGGACTAATCTTCAA	58	8041	TATACGAATGGGGGCTTCAA	58.2	120
B5	G8584A	8531	ACGAAAATCTGTTCGCTTCA	59.3	8628	GGAGGTGGGGATCAATAGAG	58.4	97

https://doi.org/10.5808/GI.2019.17.1.e11
