# Supplementary material for: Association of mitochondrial haplogroup F with physical performance in korean population
Source: Genomics Inform. 2019 Mar 31;17(1):e11. doi: 10.5808/GI.2019.17.1.e11 (PMC6459174; doi:10.5808/GI.2019.17.1.e11)
Supplement: Supplementary Table 2. — Primers for single base extension reaction [file gi-2019-17-1-e11-suppl2.pdf]

Supplementary Table 2. Primers for single base extension reaction

Haplogroup	Motif	Neutral seq	Size	Typing seq	Size	Tm(°C)	Total size
D5	A10397G	gactgactgactgactgactgactgactgactgact	36	AGTGACTACAAAAAGGATTAGACTG	25	57.5	61
F	G10310A	gactgactgactgact	16	ATGAGCCCTACAAACAACTAACCT	24	60.6	40
D	C5178a	CCCCCCCCCC	10	ACTATCTCGCACCTGAAACAAG	22	59	32
N9a	G5231A	gactgactgactgactcc	18	CCTCTCCCTAGGAGGCCT	18	59	36
D4	G3010A	gactgactgactgactgactgactgactgac	31	CGATGTTGGATCAGGACATCCC	22	61.4	53
M7	T9824C	gactgactgactgactgactgactgactgactgactga	38	CCACAGGCTTCCACGGACT	19	62.3	57
M8	A15487t	gactgactgactgactgactga	22	CTTAATGACATTAACACTATTCTCACC	27	57.3	49
A	C8794T	gactgactgactgactccccgactgactgactgact	36	GGGTGGTTGGTGTAAATGAGT	21	58.7	57
G	A4833G	gactgactgactgactccccccccccc	27	CCAGAGGTTACCCAAGGC	18	57.3	45
Y	T14178C	gactgactgactgactgactgactgactgactgactgac	39	GTTGAACATTGTTTGTTGGTGTATAT	26	57.9	65
B4,5	8281-8289d	gactgactgactgactccccccccccccc	29	ACCCTATAGCACCCCCTCTA	20	59	49
D4a	T14979C	gactga	6	CGAGACGTAAATTATGGCTGAA	22	57.2	28
D4b	G8020A	None	–	TATACGAATGGGGGCTTCAAT	21	58.7	21
N	C10873T	None	–	ACAGCCTAATTATTAGCATCATCCC	25	60.2	25
M	C10400T	None	–	TCGTTTTGTTTAAACTATATACCAATTC	28	56.3	28
N9	G5417A	None	–	CCCATATCTAACAACGTAAAAATAAAATGACA	32	60.6	32
M10	T8793C	gactgactgactgactcccc	20	CCCTCCTCGGACTCCTGCC	19	61.5	39
M9	G4491A	gactgactgactgactgact	20	CCTGCAAAGATGGTAGAGTAGATGA	25	61.3	45
M11	G11969A	gactgactgactgactgactgactgactgactgactgactgact	44	TACAGGACTCAACATACTAGTCACA	25	60.1	69
B5	G8584A	None	–	AATCCTAGGCCTACCCGCC	19	61.3	19
